# The Pharmacological Mydriatic Pupil-to-Limbal Diameter Ratio as an Intuitive Predictor for the Risk of Intraoperative Floppy Iris Syndrome

**DOI:** 10.1155/2018/2837934

**Published:** 2018-12-20

**Authors:** Yurika Terauchi, Hiroshi Horiguchi, Takuya Shiba

**Affiliations:** Department of Ophthalmology, The Jikei University School of Medicine, Tokyo, Japan

## Abstract

**Purpose:**

To predict development of intraoperative floppy iris syndrome (IFIS) using the preoperative pharmacologically dilated pupil-to-limbal diameter (PL) ratio.

**Methods:**

The subjects were male patients treated by phacoemulsification who were or were not taking *α*1-adrenoceptor antagonists (ARAs). The PL ratio was calculated from the horizontal dilated pupil diameter and the horizontal corneal white-to-white distance measured by two observers in surgical videos. IFIS severity was graded using the criteria of Chang et al. We predicted the intuitive PL ratio to describe how precisely the experimenter can estimate the PL ratio without any tools.

**Results:**

There were 36 eyes in the *α*1-ARA group and 48 eyes in the control group. The pupil diameter and PL ratio were both significantly smaller in the *α*1-ARA group compared to the control group (*p* < 0.001). All of pupil diameter, PL ratio, and intuitive PL ratio were negatively correlated with IFIS severity. The cutoff value for prediction of IFIS from the ROC curve was 7.20 mm for the pupil diameter, 58.7% for the PL ratio, and 62.5% for the intuitive PL ratio. The AUC for the ROC curve using the PL ratio (0.913) and intuitive PL ratio (0.892) did not perform substantially worse than that for the ROC curve based on the pupil diameter (0.875).

**Conclusions:**

The PL ratio is a simple and useful parameter for compensated prediction of IFIS development. Patients in whom this ratio is <60% are particularly likely to develop IFIS, and measures against onset of IFIS should be considered. This study is registered with UMIN000033012.

## 1. Introduction

Intraoperative floppy iris syndrome (IFIS) was first reported by Chang and Campbell in 2005 [1], based on an association between *α*1-adrenoceptor antagonists (ARAs) for benign prostatic hyperplasia and IFIS characterized by progressive intraoperative miosis, billowing of the iris stroma, and iris prolapse in cataract surgery. IFIS risk factors include hypertension [2–5], finasteride [3, 6–8], benzodiazepines [5], quetiapines [3, 5], alfuzosin [5, 9], doxazosin [3, 5], terazosin [5, 10], silodosin [11], and especially tamsulosin [3–5].

The incidence of IFIS was 2.3% in the initial report [1] and is 1.1% in Japan [12]. Thus, the frequency of IFIS is not high, but severe miosis or iris prolapse increases the difficulty of cataract surgery and may cause serious complications such as posterior capsule rupture and vitreous loss and iris dialysis. Accurate and convenient prediction of IFIS development before cataract surgery is critical for preparation of mechanical devices, such as iris retractors and an iris-expanding ring, before occurrence of sudden miosis and prolapse.

Chen et al. classified patients into two groups using a preoperative dilated pupil diameter of 6.5 mm and found that those with a diameter <6.5 mm had a significantly higher incidence of IFIS [13]. Similarly, Casuccio et al. found that a preoperative dilated pupil diameter <7.0 mm was a risk for IFIS regardless of treatment with *α*1-ARAs [14]. However, these previous studies needed instruments such as a pupilometer or calipers. Furthermore, pupil and cornea size are different for each person dependent on the eye size and race [15–17].

Here, we describe an intuitive and easier method for prediction of the risk for IFIS development compensated for individual eye size that does not require measurement of the pupil diameter with any tools. This study sought to assess the ratio of the pharmacological mydriatic pupil diameter relative to the corneal diameter as a useful predictor of the risk for IFIS development.

## 2. Patients and Methods

### 2.1. Patients

This study was approved by the Institutional Review Board of The Jikei University School of Medicine. We retrospectively reviewed a consecutive series of eyes treated with cataract surgery at Jikei University Daisan Hospital from May 2014 to June 2016. Only one eye for a case was included in this study. Operative records were reviewed for patients (all males) who were taking *α*1-ARAs at the time of surgery and for 48 male controls who were not taking *α*1-ARAs. The patient and control groups were age-matched and had an age range of 61–91 years old. Collected data included age, medical history, type of *α*1-ARAs, preoperative horizontal dilated pupil diameter and horizontal corneal white-to-white (WTW) distance as the limbal diameter. The exclusion criteria were previous eye surgery, traumatic cataract, and zonulopathy.

### 2.2. Measurements of the Pupil Diameter and the Pupil-to-Limbal Diameter Ratio

Approximately 90 min before surgery, mydriasis of more than a 3 times drop every 30 min was obtained using topical 0.5% phenylephrine hydrochloride and 0.5% tropicamide. To evaluate if the pupil was adequately dilated, we confirmed that the eye had no pupillary light reflex. Surgeries were performed using a 2.4 mm self-sealing temporal clear corneal or corneoscleral incision using a 2.4 mm slit knife (Alcon Laboratories, Fort Worth, TX, USA) and two side-ports using a 15 N phaco knife (Abbott Medical Optics, Santa Ana, CA, USA). A capsulorhexis was created using a cystotome needle after filling the anterior chamber with a viscoadaptive ophthalmic viscosurgical device (OVD): Opegan (Santen Pharmaceutical Co., Osaka, Japan) and/or Healon or Healon 5 (Abbott Medical Optics). Phacoemulsification (divide-and-conquer), irrigation, and aspiration were performed with Whitestar Signature® (Abbott Medical Optics) and Centurion® and Infinity® Vision systems (Alcon Laboratories) using a balanced salt solution containing Opegaurd® neo kit ocular irrigation solution (0.0184% oxiglutatione solution) (Senjyu Pharmaceutical Co., Ltd. Osaka, Japan) with 0.001% epinephrine. After a foldable intraocular lens was implanted, the incised wound was closed by stromal hydration. Surgeons chose any method, using a Healon 5 with low fluidic parameters, pupil-iris retractors, or a pupil-expanding ring. The surgeon could choose the surgical technique and use a second method if the first technique was ineffective [18].

After surgery, two observers first predicted the preoperative pupil-to-limbal diameter (PL) ratio intuitively at the beginning of surgery in anonymized surgical videos. Then, they measured the width of 2.4 mm slit knife or 15 N phaco knife and the distances between two points of corneal limbal points and pupil points in horizontal direction twice in surgical videos using a measure tool of image processing software in Microsoft PowerPoint® (Microsoft Co., Redmond, WA, USA). The dilated pupil diameter was calculated using the ratio of the pupil distance and slit knife (the width was 2.4 mm) or 15 N phaco knife (the width was 1.7 mm). The PL ratio was obtained using the ratio of corneal limbal distance and pupil distance. The timing of the dilated pupil diameter, PL ratio, and intuitive PL ratio obtained was the same when the operation was started in the videos.

Cases were classified into grades using criteria defined by Chang et al. [9]: 0, no IFIS (stable normal iris); 1, mild IFIS (noticeable iris billowing without significant miosis or iris prolapse); 2, moderate IFIS (iris billowing accompanied by iris prolapse or ≤2 mm pupil diameter reduction); 3, severe IFIS (iris billowing accompanied by iris prolapse and >2 mm pupil diameter reduction).

### 2.3. Statistical Analysis

Comparisons between the *α*1-ARA and control groups regarding dilated pupil diameters and PL ratios were performed by the Wilcoxon rank sum test. A Fisher exact test for 2 × 2 contingency tables was used to compare dilated pupil diameters and PL ratios by the presence or absence of general conditions (diabetes mellitus and hypertension) between the groups. A Steel-Dwass test as a nonparametric multiple comparison was conducted to compare the medians of all severities for dilated pupil diameter, PL ratio, and intuitive PL diameter after a Kruskal–Wallis test. Correlation coefficients among pupil diameter, PL ratio, and intuitive PL ratio were calculated using Spearman's rank correlation analysis. A Bland-Altman analysis was performed to compare the measurements of the PL ratio and the intuitive PL ratio. The limits of agreement (LoA) were defined as the mean difference plus or minus 1.96 SD of the differences (with a 95% confidence interval). The differences between the PL ratio and the intuitive PL ratio were plotted against the averages of the two methods.

To assess the predictive effect of different cutoff values for pupil size, PL ratio, and intuitive PL ratio, a receiver operating characteristic (ROC) curve was constructed and the area under the curve (AUC) was calculated, together with sensitivity, specificity, accuracy, and 95% confidence intervals (95% CIs). To obtain confidence limits on the AUCs, we fitted the data 1000 times, each fit omitting 2.5% of the data selected at random (bootstrapping method).

## 3. Results

### 3.1. Patient Characteristics

There were 36 eyes in 36 patients in the *α*1-ARA group and 48 eyes in 48 patients in the control group. The mean (±SD) age was 78.3 ± 6.8 years in the *α*1-ARA group and 77.8 ± 7.6 years in the control group (*p*=0.92; Wilcoxon rank sum test). Seven patients (19%) in the *α*1-ARA group and 14 patients (29%) in the control group had diabetes (*p*=0.44; Fisher exact test). Twenty patients (56%) in the *α*1-ARA group and 24 patients (50%) in the control group had hypertension (*p*=0.66; Fisher exact test). In the *α*1-ARA group, Grade 1 or higher IFIS was present in 24 eyes (67%): 16 (44%) in Grade 1, 4 (11%) in Grade 2, and 4 (11%) in Grade 3. In the control group, no eyes developed IFIS.

In the *α*1-ARA group, 19 patients (43%) were taking silodosin (Urief®, Kissei Pharmaceutical Co., Nagano, Japan), 16 (44%) patients were taking tamsulosin (Harnal®, Astellas Pharma Inc., Tokyo, Japan; available as Flomax® in the USA), and 5 (14%) patients were taking naftopidil (Flivas®, Asahi Kasei Pharma Co., Tokyo, Japan), an *α*1A and *α*1D antagonist that is not commercially available in the USA. One patient in the tamsulosin group and one patient in the silodosin group had a history of naftopidil treatment. Another patient had a history of treatment with all *α*1-ARAs. IFIS occurred in 15 eyes (79%) in the silodosin group, 10 eyes (63%) in the tamsulosin group, and 3 eyes (60%) in the naftopidil group, with no significant differences in incidence among the groups (*p*=0.55; Fisher exact test).

### 3.2. Comparison of Pupil Diameter, PL Ratio, and Intuitive PL Ratio

The PL ratio independent of the corneal size, which varies individually, was obtained by measurement of the pupil diameter. Using these data, the validities of the pupil diameter, PL ratio, and intuitive PL ratio for prediction of IFIS development were compared. In the *α*1-ARA and control groups, the median-dilated pupil diameters (Q1, Q3) were 6.5 (5.8, 7.3) and 7.6 (6.8, 8.4) mm and the PL ratios were 56.2 (50.1, 62.2) and 68.0 (60.0, 72.9), respectively, with both values being significantly higher in the control group (both *p* < 0.001; Wilcoxon rank sum test). In the *α*1-ARA group, the mean limbal diameter was 11.5 ± 0.6 mm, intrarater reliabilities of limbal diameters were 0.86 (95% CI, 0.75–0.93) and 0.80 (95% CI, 0.64–0.90), and interrater reliability was 0.79 (95% CI, 0.60–0.89). There was a high correlation between the pupil diameters and the PL ratios (*r* = 0.94, *p* < 0.001; Spearman's rank correlation). In the *α*1-ARA group, intrarater reliabilities of PL ratios were 0.99 (95% CI, 0.98–0.99) and 0.91 (95% CI, 0.83–0.95) and interrater reliability of PL ratios was 0.98 (95% CI, 0.96–0.99), with excellent reliabilities. The median intuitive PL ratio (Q1, Q3) in the *α*1-ARA group was 58.5 (45.8, 67.5); there was no significant difference between the PL ratio and the intuitive PL ratio (*p*=0.68; Wilcoxon rank sum test) and high correlation (*r* = 0.93, *p* < 0.001; Spearman's rank correlation). Interrater reliability of intuitive PL ratio was 0.88 (95% CI, 0.77–0.93), with almost perfect reliability.

**Figure 1:**
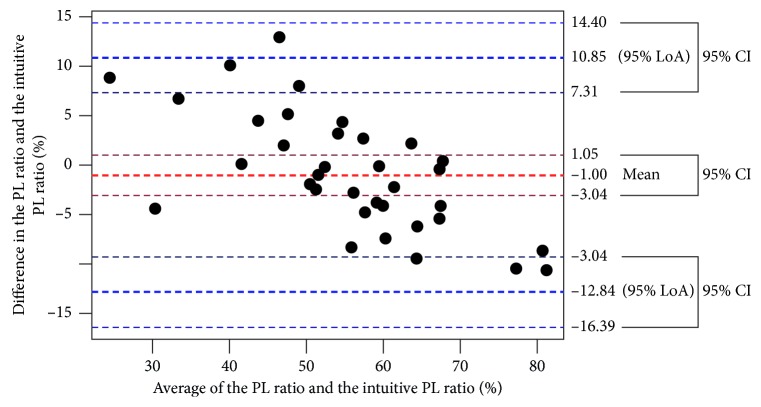
Bland–Altman plot. The difference between the PL ratio and the intuitive PL ratio is drawn against the mean of the PL ratio and the intuitive PL ratio in the 36 paired measurements. The blue dotted lines represent the LoA, and the dark blue dotted lines represent the 95% CI for the LoA.

The Bland–Altman plot is shown in Figure 1. The blue dotted lines represent the LoA (range: −12.84 to 10.85) and the dark blue dotted lines the 95% CI for the LoA. The Bland–Altman plot suggested that the measurements had a proportional error. There was a high correlation between the average and difference of the PL ratio and the intuitive PL ratio (*r* = −0.70, *p* < 0.001; Spearman's rank correlation).

In the hypertension and not having hypertension groups, the median-dilated pupil diameters were 6.4 (5.7, 7.2) mm and 6.7 (6.0, 7.3) mm (*p*=0.50; Wilcoxon rank sum test) and the median PL ratios were 53.9 (50.0, 62.2) and 58.3 (52.0, 61.4) (*p*=0.42; Wilcoxon rank sum test), with no significant difference.

The *α*1-ARA group was classified based on the grade of IFIS severity (Table 1). There was a significant difference in the preoperative dilated pupil diameter among the four groups in Table 1 (*p* < 0.01; Kruskal–Wallis test). The pupil diameter was negatively correlated with IFIS severity (*r* = −0.66, *p* < 0.001; Spearman's rank correlation). The PL ratio also differed significantly among the four groups (*p* < 0.001; Kruskal–Wallis test) and was negatively correlated with IFIS severity (*r* = −0.66, *p* < 0.001).

**Figure 2:**
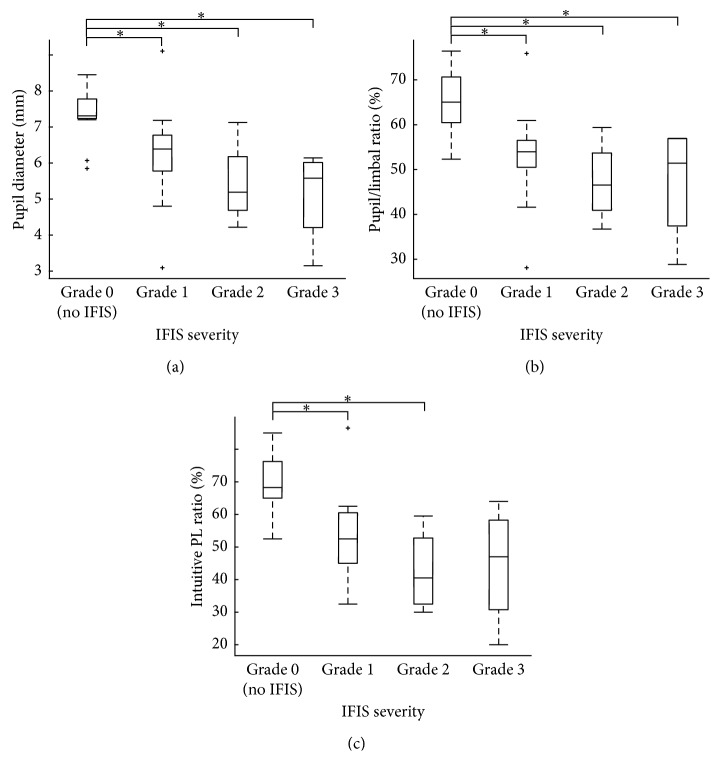
Box and whisker plot showing intraoperative floppy iris syndrome (IFIS) severity in the *α*1-ARA group. (a) IFIS severity and pupil diameter. (b) IFIS severity and the pupil-to-limbal diameter (PL) ratio. (c) IFIS severity and the intuitive PL ratio. There was significant difference between Grade 0 and not only Grade 1 but also Grades 2 and 3 in pupil diameter and PL ratio (*p* < 0.05). The intuitive PL ratio in Grade 0 cases was significantly larger than the ratios in Grade 1 and 2 cases (*p* < 0.05).

The pupil diameter and the PL ratio in Grade 0 cases were significantly larger than the ratio in Grades 1, 2, and 3 (*p* < 0.05; Steel-Dwass test) (Figure 2). There was a significant difference in the intuitive PL ratio, and the intuitive PL ratio also was negatively correlated with IFIS severity (*r* = −0.64, *p* < 0.001).

**Figure 3:**
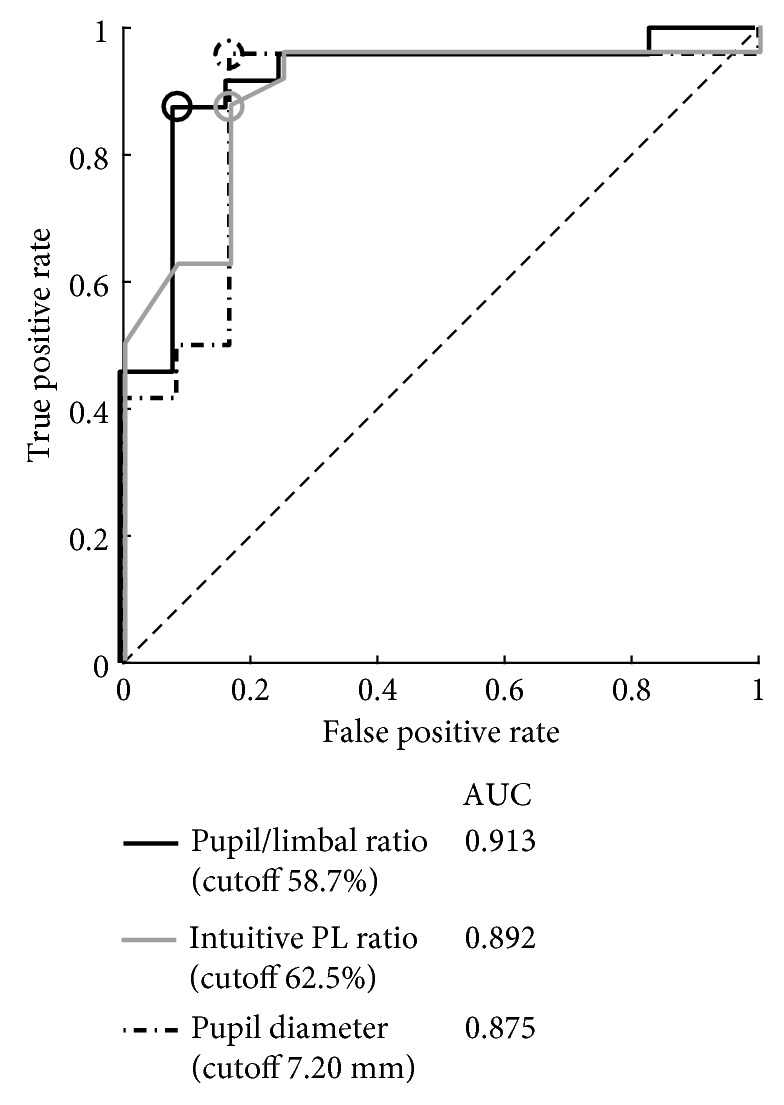
ROC curves to predict intraoperative floppy iris syndrome (IFIS). ROC curves for IFIS incidence dependent on the pupil diameter (dashed line), the pupil-to-limbal diameter (PL) ratio (solid line), and the intuitive PL ratio (gray line).

ROC curves were plotted to establish cutoff values to predict IFIS based on the pupil diameter, PL ratio, and intuitive PL ratio (Figure 3) [19]. The ROC curve analysis using the pupil diameter gave a cutoff of 7.20 mm or below, at which IFIS was predicted with 95.8% sensitivity (95% CI, 83.6%–99.9%), 83.3% specificity (95% CI, 58.9–91.2%), and 91.7% accuracy (95% CI, 75.4–96.9%). Similarly, the ROC curve analysis using the PL ratio gave a cutoff of 58.7% or less, at which IFIS was predicted with 87.5% sensitivity (95% CI, 74.8%–91.4%), 91.7% specificity (95% CI, 66.3%–99.6%), and 88.9% accuracy (95% CI, 72.0%–94.1%). The ROC curve analysis using the intuitive PL ratio gave a cutoff of 62.5% or less, at which IFIS was predicted with 87.5% sensitivity (95% CI, 74.5%–94.1%), 83.3% specificity (95% CI, 57.3%–96.6%), and 86.1% accuracy (95% CI, 68.8%–94.9%). The estimated AUCs for PL ratio, pupil diameter, and intuitive PL ratio were 0.913 (bootstrap 95% CI, 0.79–1.00), 0.875 (bootstrap 95% CI, 0.72–1.00), and 0.892 (bootstrap 95% CI, 0.76–0.99), respectively. Bootstrap analysis with 1000 replications of AUCs did not reveal any discrepancies.

### 3.3. Surgical Techniques and Complications

In the *α*1-ARA group, Healon 5 was used in 13 eyes (36%) and an iris retractor was used in one eye (3%). No pupil-expanding rings were used. Intraoperative complications occurred in 4 eyes (11%): posterior capsule rupture and vitreous loss in 3 eyes (8%) and thermal burn in one eye (3%). There were no postoperative complications.

## 4. Discussion

In this study, we examined utility of the preoperative pupil-to-limbal diameter (PL) ratio for compensated prediction of the risk for IFIS. This is the first study to examine this parameter for this purpose. The AUCs for PL ratio, pupil diameter, and intuitive PL ratio were 0.913, 0.875, and 0.892; we consider that the PL ratio and intuitive PL ratio did not perform substantially worse than pupil diameter because of the other benefit: compensated individually, preoperative, no instruments, and easy.

Thus, cataract surgeons should prepare for IFIS development in patients taking *α*1-ARA who have a PL ratio of ≤58.7%. This ratio can be estimated at a glance and without any special devices in a clinic based on a simple and intuitive method, whereas measurement of the pupil diameter requires devices such as a pupilometer or calipers. Unlike the edge of the pupil, boundaries of the limbus sometimes can be difficult to define particularly in elderly patients. We measured WTW in the horizontal direction because there were fewer errors than vertical direction due to gerontoxon and vessels.

Our results suggested a cutoff value of ≤7.20 mm for the preoperative pupil diameter for prediction of IFIS development. Casuccio et al. reported a cutoff of ≤7.0 mm for a dilated preoperative pupil diameter for prediction of the risk of severe or moderate IFIS using ROC curve analysis [14]; Chen et al. found a cutoff of ≤6.5 mm for risk of IFIS development based on classification of cases into two groups [13]; and Chang et al. proposed that a preoperative pupil diameter of ≤8.0 mm increased the risk of severe IFIS in patients taking tamsulosin, again based on classification into two groups [9]. Thus, several studies have suggested that the extent of pharmacologic pupil dilation is useful for prediction of IFIS development. The small differences in cutoff values among the current and previous studies are due to analysis methods, severity grades used to calculate ROC curves, classification criteria for severity, racial differences, and use of different *α*1-ARAs. Racial and ethnic differences affect ocular anatomy [15, 16], and the anterior segment in Asians is smaller than that in Caucasians [17]. In Japan, tamsulosin, silodosin, and naftopidil are the most prescribed *α*1-ARAs, and each have different relative receptor binding affinities: tamsulosin blocks *α*1a and *α*1d equally and with greater affinity than *α*1bARAs, silodosin blocks *α*1a > *α*1d > *α*1bARAs, and naftopidil blocks *α*1a > *α*1d = *α*1bARAs [20, 21].

The Bland–Altman plot to compare the PL ratio and the intuitive PL ratio suggested that the measurements had a proportional error. The error meant that the smaller the PL ratio was, the smaller we predicted the intuitive PL ratio and also the larger the PL ratio was, the larger we predicted the intuitive PL ratio. Especially around 60% of the average, the error was smaller. We consider that the intuitive PL ratios around the cutoff level could predict that IFIS development agrees with the actual PL ratios.

The IFIS severity grade comparisons that followed Steel-Dwass test identified a few grade comparisons which were statistically significant. However, other grade comparisons are likely different since the effect size (Δ differences) are actually larger but the sample sizes in the higher IFIS severity grades of 2 and 3 are so much smaller (only 4 patients in each of these grades).

IFIS occurred in 79% of the silodosin group, 63% of the tamsulosin group, and 60% of the naftopidil group, and these differences were not statistically significant (*p*=0.55; Fisher exact test). However, an almost 20% difference could clinically be important because the statistical test did not have strong power with the small sample sizes.

The rate of complications in this study (11%) was within the expected range, but a little higher than that found in previous studies (7–12%) [1, 22] in IFIS cases, despite the surgeons in our hospital being experienced in IFIS treatment. In particular, the incidence of posterior capsule rupture and vitreous loss was 8%. In studies reporting intraoperative complications, this incidence has ranged from 0% [14] to 12% [1]. Retrospective studies generally report higher complication rates compared to prospective studies. If surgeons could be aware of and anticipate IFIS based on *α*1-ARAs, prophylactic methods such as viscoadaptive OVD, pupil-iris retractors, or a pupil-expanding ring could be used, and this approach may decrease the rate of complications.

There are several limitations in this study. First, it was not clear if the surgeons were aware of the usage of *α*1-ARAs at the time of surgery because of the retrospective nature of the study. Second, because IFIS is subjectively diagnosed, the classification relies on an observer's subjective opinion. Third, the results may include potential grading bias, and operative records may be inadequate to classify the IFIS grade [9]. To mitigate these concerns, two observers (one operating surgeon and one who was not an operating surgeon) were masked to the allocation group when measuring the pupil and corneal diameters and predicting intuitive PL ratios on surgical videos. Fourth, because we did not completely analyze what kind of other drugs the patients were taking, the results do not apply to IFIS that develops with different medicines or conditions, and many drugs other than *α*1-ARAs, including finasteride [3, 6–8], benzodiazepines [5], and quetiapines [3, 5], might be associated with IFIS. A prospective study is required to avoid these limitations and identify a cutoff value for the PL ratio for accurate prediction of IFIS development. This is particularly important in countries with a high percentage of elderly people, who are likely to be taking *α*1-ARAs.

## 5. Conclusions

Our results suggest that the PL ratio is a simple and convenient method for compensated prediction of IFIS development without a requirement for use of measurement devices. We consider the biggest benefit of the PL ratio is that you can tell the occurrence of IFIS not during surgery but before surgery without any tools. In cataract patients with a history of taking *α*1-ARAs, we recommend that surgeons check the preoperative PL ratio and use preventive methods, such as use of a viscoadaptive OVD with low fluidic parameters, pupil-iris retractors, or a pupil-expanding ring, in patients predicted to be at risk of development of IFIS.

## Figures and Tables

**Table 1:** Dilated pupil diameters, pupil-to-limbal diameter (PL) ratios, and intuitive PL ratios (median (Q1, Q3)) with different IFIS severity grades in the *α*1-ARA group.

Parameters	Grade 0	Grade 1	Grade 2	Grade 3	*p* value^*∗*^
Eyes, *n* (%)	12 (33)	16 (44)	4 (11)	4 (11)	
Dilated pupil diameter, median (Q1, Q3)	7.3 (7.3, 7.6)	6.4 (6.0, 6.7)	5.2 (4.9, 5.7)	5.6 (4.7, 5.9)	<0.01
PL ratio, median (Q1, Q3)	65.0 (60.9, 69.0)	53.0 (50.1, 56.1)	46.6 (43.1, 50.9)	51.3 (41.7, 56.7)	<0.001
Intuitive PL ratio, median (Q1, Q3)	68.3 (66.3, 73.1)	52.5 (45.0, 60.3)	40.5 (33.8, 49.4)	47.0 (36.1, 55.4)	<0.001

^*∗*^Kruskal–Wallis test.

## Data Availability

The data used to support the findings of this study are available from the corresponding author upon request.
